# Would Older Adults with Mild Cognitive Impairment Adhere to and Benefit from a Structured Lifestyle Activity Intervention to Enhance Cognition?: A Cluster Randomized Controlled Trial

**DOI:** 10.1371/journal.pone.0118173

**Published:** 2015-03-31

**Authors:** Linda Chiu-wa Lam, Wai Chi Chan, Tony Leung, Ada Wai-tung Fung, Edward Man-fuk Leung

**Affiliations:** 1 Department of Psychiatry, The Chinese University of Hong Kong, Hong Kong; 2 Department of Psychiatry, The University of Hong Kong, Hong Kong; 3 Department of Medicine and Geriatrics, United Christian Hospital, Hong Kong; Imperial College London, UNITED KINGDOM

## Abstract

**Background:**

Epidemiologic evidence suggests that cognitive and physical activities are associated with better cognition in late life. The present study was conducted to examine the possible benefits of four structured lifestyle activity interventions and compare their effectiveness in optimizing cognition for older adults with mild cognitive impairment (MCI).

**Method and Findings:**

This was a 12-month cluster randomized controlled trial. 555 community-dwelling Chinese older adults with MCI (295 with multiple-domain deficits (mdMCI), 260 with single-domain deficit (sdMCI)) were recruited. Participants were randomized into physical exercise (P), cognitive activity (C), integrated cognitive and physical exercise (CP), and social activity (S, active control) groups. Interventions comprised of one-hour structured activities three times per week. Primary outcome was Clinical Dementia Rating sum of boxes (CDR-SOB) scores. Secondary outcomes included Chinese versions of Alzheimer’s Disease Assessment Scale - Cognitive subscale (ADAS-Cog), delayed recall, Mini-Mental State Examination, Category Verbal Fluency Test (CVFT) and Disability Assessment for Dementia – Instrumental Activities of Daily Living (DAD-IADL). Percentage adherence to programs and factors affecting adherence were also examined. At 12th month, 423 (76.2%) completed final assessment. There was no change in CDR-SOB and DAD-IADL scores across time and intervention groups. Multilevel normal model and linear link function showed improvement in ADAS-Cog, delayed recall and CVFT with time (p<0.05). Post-hoc subgroup analyses showed that the CP group, compared with other intervention groups, had more significant improvements of ADAS-Cog, delayed recall and CVFT performance with sdMCI participants (p<0.05). Overall adherence rate was 73.3%. Improvements in ADAS-Cog and delayed recall scores were associated with adherence after controlling for age, education, and intervention groups (univariate analyses).

**Conclusions:**

Structured lifestyle activity interventions were not associated with changes in everyday functioning, albeit with some improvements in cognitive scores across time. Higher adherence was associated with greater improvement in cognitive scores. Factors to enhance adherence should be specially considered in the design of psychosocial interventions for older adults with cognitive decline.

**Trial Registration:**

ClinicalTrials.gov ChiCTR-TRC-11001359

## Introduction

It is estimated that 35.6 million people lived with dementia worldwide in 2010, and the number will double every 20 years.[1] The rising prevalence has made prevention of neurodegenerative dementias a major public health concern. No pharmacological treatment, including cholinesterase inhibitors, has been found to be effective in delaying the onset of dementia at present.[2,3] It is therefore not surprising to see an increasing interest in the research on modifiable lifestyle factors that are suggested by epidemiological data to be potentially protective against cognitive decline.[4–9] The number of randomized controlled trials examining the efficacies of lifestyle interventions in improving cognitive function has remained limited, partly because of the methodological complexity in study design.[10]

Evidence shows that a higher level of lifestyle activity participation, especially cognitive and physical activities, may help to preserve cognition at late life. The beneficial effects of physical exercise may act through fostering cardiovascular fitness with improvement in cerebral circulation, and enhancing neuroplastic responses through physiological changes that involves the brain-derived neurotrophic factor. Similarly, cognitive activity participation is associated with better cognition, which is also postulated to be related to neuroplasticity changes and neuronal stimulation in relation to activities that demands high cognitive load.[4–10] Interestingly, the protective effect of these activities appears to be independent of known pathological burden of Alzheimer’s disease, which opens up an option for adjuvant interventions that act through pathways other than the amyloid or tau pathologies.[11] While the findings are encouraging, it is not easy to administer lifestyle interventions to promote cognitive health in older adults.[12] Firstly, alterations in lifestyles are hard to achieve, and even more difficult to sustain. This becomes especially challenging to people who have already experienced cognitive decline. Secondly, though laboratory-based paradigms of cognitive training may bring about positive benefits, they are frequently abandoned upon completion of the clinical trials and are difficult to be translated into lifestyle changes. Thirdly, there is limited information as to how and which lifestyle activities may benefit cognition at different stages of impairment. It is therefore difficult for clinicians to recommend appropriate activities for clients seeking advice on lifestyle modifications that may enhance cognitive health.

The present study explored the above issues with a cluster randomized controlled trial (RCT) of a structured lifestyle activity intervention in Chinese older adults with mild cognitive impairment (MCI). In this study, we organized the activity interventions, which comprised a selection of leisure activities indigenous to the local community, with simple structured schedules. Based on available research findings of physical exercise and cognitive activities, four intervention groups with activity schedules consisting of Cognitive (C), Physical (P), integrated Cognitive-Physical (CP) and Social (S) activity programs were designed. The S group served as active control for the nonspecific effects of psychosocial intervention. We hypothesized that single modality C or P programs would be associated with better cognitive and functional outcomes over S program, and dual modality CP program would be better in efficacy over the C or P programs. The four intervention groups were compared for their cognitive and functional outcomes. Factors associated with adherence to programs, and the impacts of adherence on cognitive outcomes, were also evaluated.

## Methods

### Ethics Approval

The study obtained approvals from the Survey and Behavioral Research Ethics Committee (SBREC 28 March 2011), and the Joint CUHK-NTEC Clinical Research Ethics Committee of the Chinese University of Hong Kong (5 May 2011). Written informed consent was obtained from all participants before commencement of study. The first group of subject recruitment started in April 2011 after obtaining ethics approval from SBREC and registration with the university clinical trial registry (CUHK CCT Clinical Trials Registry). The trial was registered under the Chinese Clinical Trial Registry CLINICAL TRIAL IDENTIFIER: ChiCTR-TRC-11001359 (http://www.chictr.org/en/proj/show.aspx?proj=47). The authors confirm that all ongoing and related trials for this intervention are registered. The protocol for this trial and supporting CONSORT checklist are available as supporting information (See S1 CONSORT Checklist and S1 Protocol).

### Design

This study adopted a single blind cluster RCT design of 12-month duration. Participant recruitment started in April 2011 till August 2011. Intervention started in July 2011, and the last observations were completed in September 2012.

### Recruitment centers and participants

Participants (aged 60 years or over) were recruited through social centers for elders through three non-governmental organizations in Hong Kong. Eligible participants should satisfy all of the following criteria:

1. Participants should have mild cognitive impairment (MCI) as defined by the presence of subjective cognitive complaints, and objective impairments in cognitive function. The impairment of episodic memory was set at > = 1.5 standard deviation (SD) below education- and age-matched normal subjects.[13] Non-memory cognitive domains included category verbal fluency test, attention span. Impairments for MCI in these domains were set at > = 1 SD below matched norms according to a previous study of epidemiological sample.[14] Participants were categorized into MCI-single domain deficit (sdMCI) or MCI-multiple domain deficits (mdMCI) according to their baseline cognitive performance. The sdMCI group exhibited deficit in only one cognitive domain (memory or non-memory) was impaired. The mdMCI group had impairments in more than one cognitive domain.
2. Participants should be physically stable as assessed by the psychiatrist of the research team.

The exclusion criteria included a diagnosis of dementia and CDR > = 1,[15] concurrent treatment with anti-dementia medications or has been receiving other types of cognitive training at the time of study. Participants who did not attend the activity assigned due to physical health reasons, lack of interests or moving out of the usual place of residence, were considered as dropouts.

### Randomization and Group Assignment

Participants were recruited and allocated into activity groups (size 12 to 15) in social centers near to their place of residence. The activity groups were then randomly assigned to different intervention (P, C, CP, S) groups in a block of 4 to ensure a balance in assignment of interventions. The PI (LCWL) generated the randomization code for each center. Participants were not stratified according to cognitive status (sdMCI and mdMCI). Designation of sdMCI and mdMCI was made according to the baseline cognitive status after randomization of intervention program.

Interventions were conducted in groups at the participating social centers, and supplemented by home-based activities. Thirty-three lifestyle leisure activities commonly endorsed by Chinese older adults were categorized into cognitive, physical, social and recreational activities according to a lifestyle activity reference list recently developed through focused group discussions and survey of cognitively healthy Chinese older adults in Hong Kong (S1 Appendix).[16] Cognitive activities were leisure activities with consensus of higher demands on cognitive efforts (e.g. reading, calligraphy, playing a musical instrument). Physical exercises refer to aerobic exercise, mind body exercise, resistance training, stretching and toning (e.g. cycling, brisk walking, Tai Chi). Social activities refer to activities having a social component but with low physical or cognitive demands (e.g. tea gathering or shopping with friends). For each intervention program, activities were selected from the corresponding category with the following schedule:

1. The Social (S) group attended a selection of social activities from the reference list (e.g. tea gathering, film watching). The participants were arranged to attend at least three one-hour social activity sessions per week. It served as the active control arm.
2. The Cognitive (C) group attended cognitively demanding activities selected from the reference list (e.g. reading and discussing newspapers, playing board games). In each week, at least three cognitive activity sessions were arranged.
3. The Physical (P) group was arranged to have physical exercise practice. The exercise schedule comprised one stretching & toning exercise, one mind body exercise (e.g. Tai Chi) and one aerobic exercise session (e.g. static bicycle riding) in a week. Each exercise session lasted for an hour;
4. The integrated Cognitive-Physical (CP) group took part in one cognitive and two types of mind body exercise. For any week, three one-hour sessions were arranged.

For centers with few participants, one intervention group was assigned. For centers with more participants, more than one intervention groups were assigned. The allocation of intervention groups was randomized according to the timing of recruitment of subjects. At any participating center, special attention in the allocation of program schedule was made to avoid communication among participants from different groups throughout the training period. The assessors for clinical outcomes were research assistants with no involvement in the intervention programs. They carried out outcome assessments as scheduled by center staffs and were blind to the randomization status.

### Intervention and assessment schedule

For each intervention program, staff members at the social centers provided supervised training of the designated activities to participants during the study period. Participants were required to carry out activities at the centers at least once per week. They were encouraged to continue the remaining activities at the center, or carried out activities at home under supervision by family members. The center staffs kept track of the attendance and logged program adherence. If a participant failed to turn up at the training center, the staffs would contact the participants and family members to ensure that the practice reached the recommended frequency. Cognitive and functional assessments were conducted at the baseline, 4th, 8th and 12th months.

### Assessment tools and clinical outcomes

#### Primary outcome

1. Clinical Dementia Rating sum of boxes (CDR-SOB)[15]—CDR is a semi-structured clinical interview for assessment of global cognitive ability. As global CDR score is limited in the range of assessment (0 to 3), and ceiling effects are expected for participants with no dementia, we adopted the CDR-SOB as the primary outcome for observe evaluation of global cognition and functioning. The CDR-SOB refers to summative scores of the six domains (orientation, memory, judgment and problem-solving, home and hobbies, personal care, and community affairs). A higher score indicates more severe impairment.

#### Secondary outcomes

1. A cognitive battery comprised of the Cantonese version of the Alzheimer’s Disease Assessment Scale—Cognitive subscale (ADAS-Cog).[17] ADAS-Cog is a global cognitive assessment scale sensitive to changes characteristics of early Alzheimer’s disease (range 0 to 70). It includes subtests on episodic memory, agnosia, ideational apraxia, visuospatial construction, orientation and recognition. A higher ADAC-Cog score indicates more severe cognitive impairment. The Cantonese version of Mini-Mental State Examination (CMMSE) is a standard screening test to reflect global cognitive function with higher scores indicating better cognitive function.[18] Apart from tests on global cognitive function, the other test included the list learning delayed recall test for episodic memory, digit and visual span test for attention and working memory, category verbal fluency test (CVFT)[19] and Chinese trail making test. Subjective cognitive complaints (Memory Inventory for Chinese, MIC).[20] MIC is a questionnaire enquiring memory and other related cognitive complaints previously validated in Chinese older adults in Hong Kong. A higher score indicates more cognitive complaints.
2. The Cornell Scale for Depression in Dementia (CSDD) was used to assess depressive symptoms.[21] It is an observer rated instrument to track the severity of depression in older adults with cognitive impairment. A higher score indicates more severe depression and a cutoff of 6 indicated clinically significant depression in Chinese older adults.
3. The instrumental activity of daily living (IADL) is measured by the Chinese Disability Assessment for Dementia (CDAD).[22] CDAD is a locally validated functioning assessment for older adults with different degree of cognitive abilities. It has 4 questions on basic ADL and 7 items on IADL (meal preparation, use of device, transportation, money handling, and medication management). For each item, three dimensions on initiation, organization and effectiveness to carry out a specific task would be measured to give an overall rating. As participants were not clinically demented upon recruitment, they have independent basic ADL, so only IADL terms of the CDAD were analyzed.
4. Adherence rates were assessed by percentage participation of sessions per week. In the estimation of % adherence, activities performed both at centers and homes were counted. If a participant’s adherence to the 12-month schedule was over 70%, adherence was considered as ‘satisfactory’.

### Sample size estimation and statistical analyses

The sample size was estimated with a cluster-randomization design. Participants were recruited into activity group at the participating centers, which were then randomized into different intervention programs. Measurements were carried out at multiple levels (intervention programs, activity groups and person). With three repeated measurements (baseline, 4th, 8th, and 12th month) and assuming a small treatment by time interaction effect compared with the S (control) program (Cohen’s d = 0.30), eight groups per intervention (C, P, CP, S) and 15 participants per group would be adequate to achieve a power of 0.8 with an alpha of 0.05. Taking into account of an estimated dropout rate of about 30%, 11 groups per arm were recruited.[23,24]

Cognitive and everyday function was compared between different intervention groups with a cluster randomization approach. Intention—to-treat analysis was carried out for participants who had completed baseline assessment. Baseline differences between intervention (C, P, CP) and control (S) groups were evaluated with a two-level model with subjects at level one and activity groups at level two. All available observations were analyzed and subjects were not removed even if data at one time point was missing. The mean efficacy parameters (CDR-SOB, cognitive and everyday functioning, mood symptom) for each intervention group were computed for each visit. Multilevel generalized linear modeling was employed to account for the correlations within subjects and within activity groups. The model allowed for missing values occurred in between time points.

Changes of efficacy indicators from baseline to each follow up point and intervention group differences were tested with a three-level model with occasions (time points) at level one, subjects at level two, and activity groups at level three. Response variables with normal distribution (cognitive test scores) were tested with the multilevel normal model and linear link function. For response variables (CSDD, CDR-SOB) that were skewed in distribution, they were tested with the multilevel Poisson model and log link function. To explore if the intervention might exert different effects in participants at different severity of cognitive impairment, secondary post-hoc analyses were carried out with comparisons of outcomes in sdMCI and mdMCI with the above-mentioned procedure respectively.

Adherence (%) was compared among different activity schedules. For cognitive scores that showed changes with time, the association between % program adherence and improvement in scores was analyzed by univariate analysis controlling for other factors that may influence cognitive outcome. Statistical analyses were performed using STATA version 12.0 and IBM SPSS 20.0. Significance level was set at p< 0.05.

## Results

### Baseline characteristics

**Fig 1:**
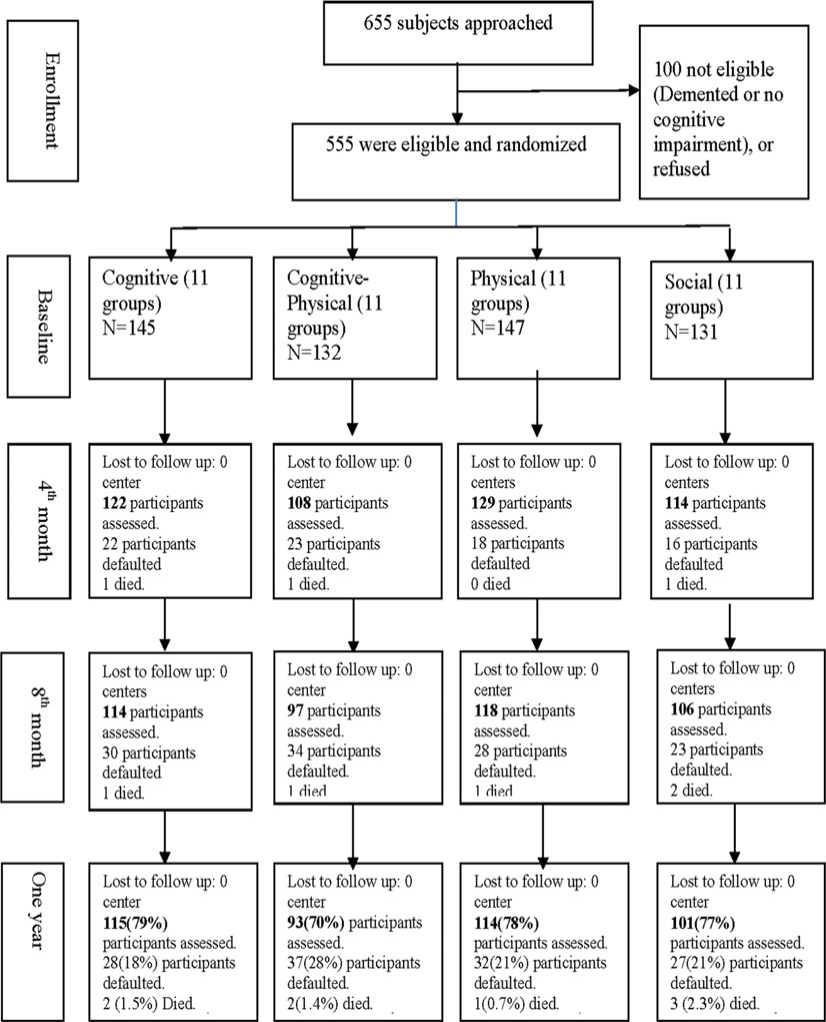
CONSORT flow diagram.

Six hundred fifty-five participants were recruited for baseline assessment. Among them, 100 participants were considered not eligible (dementia with CDR> = 1, no cognitive deficits, declined participation due to inability to match schedules of intervention). 555 subjects were randomized into four intervention groups (C = 145, CP = 132, P = 147, S = 131), with 260 participants belonged to sdMCI and 295 mdMCI categories. The average group size was 12 to 13. Fig. 1 depicted flow of participants from screening through study completion. Of the 555 participants, 434 were women (78.2%), the mean age and education level was 75.4 (6.5) years and 3.9 (3.6) years. There were no group differences in age, educational level, gender, proportion of sdMCI and mdMCI, cognitive characteristics and mood symptoms at baseline (Multilevel model, Table 1). Cognitive characteristics of the sdMCI and mdMCI participants were presented in Table 2.

**Table 1:** Baseline demographic and cognitive characteristics of intervention groups.

	Cognitive(Baseline N = 145)	Cognitive-Physical(Baseline N = 132)	Physical(Baseline N = 147)	Social(BaselineN = 131)
**Age**	74.4(6.4)	76.3(6.6)	75.5(6.7)	75.4(6.1)
**Education (in yrs)<Median, Range>**	3.9(3.8)<3, 0–15>	3.4(3.3)<3, 0–17>	4.0(3.6)<4, 0–15>	4.0(3.9)<3, 0–17>
**Gender (M:F)**	30:115	28:104	34:113	29:102
**sdMCI: mdMCI**	70:75	59:73	75:72	56:75
**CDR-SOB<Median, Range>**	0.9(0.6)<1, 0–2.5>	1.0(0.6)<1, 0–2>	0.9(0.6)<1, 0–2.5>	1.0(0.7)<1, 0–3>
**CMMSE**	25.7(2.4)	25.2(2.2)	25.8(2.3)	25.6(2.4)
**ADAS-Cog**	11.3(3.2)	11.6(3.4)	11.7(3.3)	11.5(3.4)
**Delayed Recall**	3.5(2.3)	3.2(2.2)	3.5(2.3)	3.4(2.1)
**CVFT**	34.2(7.3)	32.8(6.7)	33.3(7.3)	32.7(7.4)
**Digit Span (forward)**	7.1(1.2)	6.5(1.4)	6.7(1.4)	6.7(1.5)
**Digit Span (Backward)**	3.7(2.2)	3.5(1.6)	3.6(1.8)	3.6(1.8)
**Trail A (in seconds)**	26.1(24.5)	27.0(17.8)	25.1(18.4)	27.0(17.5)
**Trail B (in seconds)**	130.0(67.4)	137.2(70.7)	124.1(61.1)	131.2(70.1)
**SCC**	7.5(4.3)	6.9(4.3)	7.3(4.4)	8.2(4.5)
**DAD-IADL**	0.95(0.08)	0.97(0.06)	0.98(0.05)	0.95(0.07)
**CSDD (total)<Median, Range>**	0.6(2.2)<0, 0–19>	0.8(2.4)<0, 0–21>	0.7(2.6)<0, 0–24>	0.7(1.9)<0, 0–17>

()—Standard deviation, sdMCI—Mild Cognitive Impairment, single domain deficit, mdMCI- Mild Cognitive Impairment, multiple domain deficit; CDR-SOB—Clinical Dementia Rating sum of boxes; CMMSE—Cantonese version of Mini-mental state examination; CVFT—category verbal fluency test; SCC—Subjective Cognitive Complaints; DAD-IADL—Instrumental activities of daily living of the Chinese Disability Assessment for Dementia; CSDD—Cornell Scale for Depression in Dementia. Group comparisons—Multi-level linear model-Baseline differences between intervention groups were evaluated with two-level model with subjects at level one and activity groups at level two. Differences between intervention groups were not significant.

**Table 2:** Demographic and baseline cognitive profiles of sdMCI and mdMCI participants.

	sdMCI(N = 260)	mdMCI(N = 295)	Comparisons(p value)
Age	74.2(6.4)	76.4(6.4)	4.01(<0.001)
Education (in years)<Median, Range>	4.1(3.8)<3, 0–17>	3.6(3.5)<3, 0–15>	1.71(0.09)
CDR-SOB<Mean, Range>	0.6(0.5)<0.5, 0–2>	1.3(0.6)<1.5, 0–3>	13.80(<0.001)
CMMSE	26.8(1.8)	24.6(2.3)	12.66(<0.001)
ADAS-Cog	9.5(2.5)	13.3(2.9)	16.40(<0.001)
Delayed Recall	4.7(1.9)	2.3(1.8)	15.29(<0.001)
CVFT	35.9(6.4)	30.9(7.0)	8.67(<0.001)
Digit Span (Forward)	6.9(1.3)	6.6(1.4)	3.23(<0.001)
Digit Span (Backward)	2.6(1.1)	2.3(1.1)	3.96(<0.001)
Trail A (in Seconds)	22.1(14.4)	29.9(23.0)	4.82(<0.001)
Trail B (in Seconds)	115.2(60.6)	149.3(70.4)	4.99(<0.001)
CSDD<Median, Range>	0.6(1.6)<0, 0–17>	0.8(2.7)<0, 0–24>	1.2(0.23)

()—Standard deviation, sdMCI—Mild Cognitive Impairment, single domain deficit, mdMCI- Mild Cognitive Impairment, multiple domain deficit; CDR-SOB—Clinical Dementia Rating sum of boxes; CMMSE—Cantonese version of Mini-mental state examination; CVFT—category verbal fluency test; SCC—Subjective Cognitive Complaints; CSDD—Cornell Scale for Depression in Dementia. Comparisons (t-tests)

### Study Outcomes at study end

At 12^th^ month, 423 (76%) completed the intervention. Eight (1.4%) participants died of physical illnesses during the study period. There were no reported adverse events associated with the intervention. No difference in age and education were observed between completers and defaulters. Defaulters had lower delayed recall (p = 0.007) and lower CSDD scores (p = 0.04) at baseline. Eighteen participants (3.2%, C = 5, CP = 5, P = 3, S = 5) progressed to dementia at one year.

There were no change in CDR-SOB (p = 0.92), CDAD-IADL (p = 0.15) and CMMSE scores (p = 0.16) at the end of study. Improvements in digit forward span (Multi-level model, p = 0.005), ADAS-Cog, delayed recall, CVFT were observed in all groups over time (Multi-level model, p< 0.001). No intervention group differences were observed. Digit backward span, CDAD-IADL and trail making tests showed no time or intervention group differences (Multi-level model). The CP group showed greater improvements in CVFT (time × intervention effects, χ^2^ = 23.38, p< 0.001). There was a decrease in subjective cognitive complaints (MIC) and depressive symptoms (CSDD) across all groups with time (p<0.001) (Table 3).

**Table 3:** Effects of activity intervention on cognitive and mood outcomes for all participants (Intention-to-treat method).

	Intervention groups					Change over time (Chi square)	Intervention by time (Chi square)
	Cognitive(Baseline n = 145)	Cognitive-Physical(Baseline n = 132)		Physical (Baseline n = 147)	Social(Baseline n = 131)		
**CDR-SOB**						0.01(p = 0.92)	1.83(p = 0.61)
**Baseline**	0.9(0.6)	1.0(0.7)	0.9(0.6)		1.0(0.7)		
**4** ^**th**^ **month**	0.8(0.6)	0.8(0.6)	0.7(0.6)		0.9(0.6)		
**8** ^**th**^ **month**	0.8(0.7)	1.1(0.7)	0.8(0.6)		1.0(0.8)		
**12** ^**th**^ **month**	0.8(0.7)	1.0(0.8)	0.8(0.6)		1.0(0.7)		
**CMMSE**						1.97(p = 0.16)	4.28(p = 0.23)
**Baseline**	25.7(2.4)	25.2(2.3)	25.8(2.3)		25.6(2.4)		
**4** ^**th**^ **month**	25.8(2.6)	25.7(2.5)	26.2(2.2)		25.8(2.4)		
**8** ^**th**^ **month**	25.2(2.6)	25.2(2.6)	26.0(2.1)		25.8(2.2)		
**12** ^**th**^ **month**	25.2(2.9)	25.2(2.9)	25.6(2.4)		25.5(2.5)		
**ADAS-Cog**						91.42(p<0.001)	4.65(p = 0.20)
**Baseline**	11.3(3.2)	11.6(3.4)	11.7(3.3)		11.5(3.4)		
**4** ^**th**^ **month**	8.8(3.5)	8.9(3.2)	8.8(3.6)		9.2(3.3)		
**8** ^**th**^ **month**	8.8(3.9)	8.4(3.4)	9.4(3.8)		9.0(3.6)		
**12** ^**th**^ **month**	8.0(3.4)	7.9(3.6)	8.4(3.3)		8.4(3.3)		
**Delayed Recall**						52.17(p<0.001)	3.31(p = 0.35)
**Baseline**	3.5(2.2)	3.2(2.2)	3.5(2.3)		3.4(2.1)		
**4** ^**th**^ **month**	5.8(2.1)	5.3(2.1)	5.7(2.3)		5.4(2.1)		
**8** ^**th**^ **month**	4.8(2.2)	4.5(2.2)	4.5(2.2)		4.4(2.2)		
**12** ^**th**^ **month**	5.8(2.3)	5.4(2.2)	5.3(2.4)		5.6(2.2)		
**CVFT**						11.54(p = <0.001)	23.38(p = <0.001)
**Baseline**	34.2(7.3)	32.8(6.7)	33.3(7.3)		32.7(7.4)		
**4** ^**th**^ **month**	36.2(8.2)	35.8(7.2)	35.7(8.0)		34.4(7.9)		
**8** ^**th**^ **month**	36.7(8.7)	38.2(7.4)	33.6(7.2)		34.2(8.4)		
**12** ^**th**^ **month**	36.5(8.6)	36.9(8.1)	34.3(8.0)		35.5(7.8)		
**Subjective cognitive complaints**						33.42(p<0.001)	1.65(p = 0.65)
**Baseline**	7.5(4.3)	6.9(4.3)	7.3(4.4)		8.2(4.5)		
**4** ^**th**^ **month**	6.7(4.0)	6.5(3.7)	7.2(3.9)		7.6(4.1)		
**8** ^**th**^ **month**	6.0(3.7)	5.8(3.3)	6.2(3.6)		7.1(3.6)		
**12** ^**th**^ **month**	5.5(3.0)	5.7(3.0)	5.6(3.7)		6.6(3.9)		
**CDAD-IADL**						2.04(p = 0.15)	1.0(p = 0.80)
**Baseline**	0.95(0.08)	0.97(0.06)	0.98(0.05)		0.95(0.07)		
**4** ^**th**^ **month**	0.97(0.05)	0.98(0.08)	0.97(0.04)		0.96(0.06)		
**8** ^**th**^ **month**	0.96(0.07)	0.95(0.06)	0.97(0.05)		0.98(0.07)		
**12** ^**th**^ **month**	0.95(0.07)	0.94(0.08)	0.96(0.06)		0.94(0.07)		
**CSDD (total)**						26.29(p = <0.001)	0.85(p = 0.84)
**Baseline**	0.6(2.2)	0.8(2.4)	0.7(2.6)		0.7(1.9)		
**4** ^**th**^ **month**	0.3(1.2)	0.2(0.8)	0.1(0.6)		0.4(1.2)		
**8** ^**th**^ **month**	0.1(0.6)	0.2(0.4)	0.2(0.6)		0.2(0.5)		
**12** ^**th**^ **month**	0.1(0.4)	0.1(0.6)	0.1(0.4)		0.1(0.5)		

() Standard Deviation, CDR-SOB—Clinical Dementia Rating sum of boxes; CMMSE—Cantonese version of Mini-mental state examination; ADAS-Cog—Alzheimer's Disease Assessment Scale cognitive subscale; CVFT—category verbal fluency test; SCC—Subjective Cognitive Complaints; CSDD—Cornel Scale for Depression in Dementia. Multi-level model-Differences between intervention groups were evaluated with three-level model with time point at level one, subjects at level two and activity groups at level three.

### Subgroup analyses (sdMCI and mdMCI) of cognitive outcomes at one year

Post-hoc subgroup analyses were carried out for the sdMCI and mdMCI groups. There was no change in CDR-SOB. Improvements in ADAS-Cog, delayed recall, subjective cognitive complaints (MIC) and CVFT were found in both groups with time (p< 0.001). For the sdMCI group, a time trend for lower CMMSE scores was observed (p = 0.04). In sdMCI group, integrated CP intervention were associated with better performance in ADAS-Cog (time × group effects, χ^2^ = 10.25, p = 0.02), CVFT (time × group effects, χ^2^ = 12.41, p = 0.006) and delayed recall (time × group effects, χ^2^ = 9.45, p = 0.02) than other interventions (Table 4). For the mdMCI participants, the CP group was associated with greater improvement in CVFT (time × group effects, χ^2^ = 11.64, p = 0.009), with no group differences observed in other cognitive tests (Table 5).

**Table 4:** Effects of activity intervention on cognitive and mood outcomes for participants with MCI-single domain deficit (Intention-to-treat method).

	Intervention groups				Change over time (Chi square)	Intervention by time (Chi square)
	Cognitive(Baseline n = 70)	Cognitive-Physical(Baseline n = 59)	Physical (Baseline n = 75)	Social(Baseline n = 56)		
**CDR-SOB**					1.5(p = 0.22)	1.67(p = 0.64)
**Baseline**	0.6(0.5)	0.6(0.5)	0.6(0.6)	0.6(0.6)		
**4** ^**th**^ **month**	0.5(0.5)	0.7(0.7)	0.4(0.5)	0.7(0.6)		
**8** ^**th**^ **month**	0.6(0.6)	0.8(0.6)	0.5(0.6)	0.7(0.7)		
**12** ^**th**^ **month**	0.5(0.6)	0.7(0.6)	0.6(0.6)	0.8(0.7)		
**CMMSE**					4.05(p = 0.04)	2.46(p = 0.48)
**Baseline**	27.0(1.8)	26.5(1.8)	26.9(2.0)	26.7(1.7)		
**4** ^**th**^ **month**	27.0(2.1)	26.7(2.0)	26.9(1.9)	26.6(1.9)		
**8** ^**th**^ **month**	27.1(2.0)	26.2(2.2)	27.0(1.8)	26.6(1.9)		
**12** ^**th**^ **month**	26.9(2.3)	26.1(2.3)	26.4(2.0)	26.2(2.1)		
**ADAS-Cog**					31.06(p< 0.001)	10.25(p = 0.02)
**Baseline**	9.5(2.3)	9.8(2.9)	9.5(2.5)	9.2(2.5)		
**4** ^**th**^ **month**	6.8(2.1)	7.4(2.3)	6.9(2.7)	7.7(2.4)		
**8** ^**th**^ **month**	6.9(2.4)	6.9(3.0)	7.4(2.9)	7.4(2.6)		
**12** ^**th**^ **month**	6.4(2.3)	6.1(2.7)	7.2(2.9)	7.2(2.9)		
**Delayed Recall**					15.91(p< 0.001)	9.45(p = 0.02)
**Baseline**	4.7(2.1)	4.3(2.1)	4.9(1.9)	4.9(1.6)		
**4** ^**th**^ **month**	6.7(1.6)	6.0(2.2)	6.6(1.8)	5.8(2.1)		
**8** ^**th**^ **month**	5.6(2.1)	5.3(2.1)	5.2(2.0)	5.3(2.0)		
**12** ^**th**^ **month**	6.8(1.8)	6.7(1.8)	6.1(2.2)	6.5(2.0)		
**CVFT**					5.30(p = 0.02)	12.41(p = 0.006)
**Baseline**	37.5(6.5)	35.0(5.8)	35.7(6.6)	35.3(6.5)		
**4** ^**th**^ **month**	39.7(7.9)	38.7(6.5)	38.1(7.8)	36.7(7.6)		
**8** ^**th**^ **month**	40.5(7.6)	40.2(6.5)	36.3(7.1)	36.5(7.3)		
**12** ^**th**^ **month**	39.7(7.9)	39.7(7.9)	36.9(8.1)	37.2(7.1)		

() Standard Deviation, CDR-SOB—Clinical Dementia Rating sum of boxes; CMMSE—Cantonese Mini-mental state examination; ADAS-Cog—Alzheimer's Disease Assessment Scale cognitive subscale; CVFT—category verbal fluency test;. Differences between intervention programs were evaluated with three-level model with time point at level one, subjects at level two and activity groups at level three.

**Table 5:** Effects of activity intervention on cognitive and mood outcomes for participants with MCI-multiple domains deficits (Intention-to-treat method).

	Intervention groups				Change over time 9Chi square)	Intervention by time (Chi square)
	Cognitive(Baseline n = 75)	Cognitive-Physical(Baseline n = 73)	Physical (Baseline n = 72)	Social(Baseline n = 75)		
**CDR-SOB**					0.57(p = 0.45)	1.6(p = 0.66)
**Baseline**	1.3(0.5)	1.3(0.6)	1.2(0.5)	1.3(0.6)		
**4** ^**th**^ **month**	1.1(0.6)	1.0(0.6)	1.0(0.5)	1.1(0.6)		
**8** ^**th**^ **month**	1.1(0.6)	1.3(0.6)	1.0(0.6)	1.2(0.8)		
**12** ^**th**^ **month**	1.2(0.8)	1.4(0.8)	1.1(0.6)	1.1(0.7)		
**CMMSE**					0.01(p = 0.91)	2.43(p = 0.49)
**Baseline**	24.5(2.2)	24.2(2.0)	24.7(2.2)	24.8(2.5)		
**4** ^**th**^ **month**	24.7(2.4)	24.7(2.5)	25.4(2.2)	25.1(2.5)		
**8** ^**th**^ **month**	25.0(2.6)	24.3(2.7)	25.0(1.9)	25.2(2.4)		
**12** ^**th**^ **month**	24.9(2.5)	24.3(3.0)	24.7(2.4)	24.9(2.7)		
**ADAS-Cog**					60.2(p< 0.001)	0.21(p = 0.98)
**Baseline**	13.1(3.0)	13.1(3.1)	13.9(2.4)	13.1(2.9)		
**4** ^**th**^ **month**	10.7(3.6)	10.2(3.3)	10.7(3.3)	10.5(3.4)		
**8** ^**th**^ **month**	11.0(4.1)	9.8(3.3)	11.6(3.4)	10.4(3.8)		
**12** ^**th**^ **month**	9.7(3.6)	9.6(3.6)	9.9(3.0)	9.6(3.3)		
**Delayed Recall**					37.49(p< 0.001)	0.54(p = 0.91)
**Baseline**	2.4(1.8)	2.4(2.0)	2.0(1.7)	2.3(1.8)		
**4** ^**th**^ **month**	4.9(2.1)	4.8(1.9)	4.7(2.3)	5.0(2.1)		
**8** ^**th**^ **month**	4.0(2.0)	3.8(2.1)	3.7(2.2)	3.6(2.1)		
**12** ^**th**^ **month**	4.6(2.2)	4.2(1.9)	4.4(2.3)	4.8(2.1)		
**CVFT**					6.71(p = 0.01)	11.64(p = 0.009)
**Baseline**	31.1(6.6)	31.0(6.9)	30.9(7.3)	30.8(7.4)		
**4** ^**th**^ **month**	33.0(7.3)	33.5(6.9)	33.3(7.5)	32.5(7.6)		
**8** ^**th**^ **month**	32.4(7.8)	36.4(7.7)	30.6(6.1)	32.1(8.8)		
**12** ^**th**^ **month**	33.0(7.3)	34.2(7.4)	31.3(6.9)	33.9(8.2)		

() Standard Deviation, CDR-SOB—Clinical Dementia Rating sum of boxes; CMMSE—Cantonese Mini-mental state examination; ADAS-Cog—Alzheimer's Disease Assessment Scale cognitive subscale; CVFT—category verbal fluency test;. Differences between intervention programs were evaluated with three-level model with time point at level one, subjects at level two and activity groups at level three.

### Program adherence & factors associated with changes in cognitive function

The overall adherence rate was 73% (i.e. over 70% attendance to activity schedule throughout the 12-month period), with the lowest in the CP group (P, 75%; C, 75%; CP, 65%; S, 71%) (One way ANOVA, p = 0.03). While age and education had no association with % adherence, women exhibited a higher adherence (t-test, p = 0.007). There was a weak positive correlation between better baseline MMSE (r = 0.09) and delayed recall (r = 0.09) scores with % adherence (Pearson correlations, p< 0.05). There were significant improvements in ADAS-Cog (Mean change, 95% CI = 3.04, 2.77–3.31) and delayed recall (Mean change, 95% CI = 1.95, 1.75–2.16) scores at the end of the study. A higher % adherence to program was associated with improvement in ADAS-Cog scores (Univariate analysis, p< 0.001). Improvement in delayed recall scores was associated with younger age (p<0.001), baseline sdMCI group (p<0.001) and higher % adherence to program (Univariate analysis, p = 0.008). Improvement in CVFT (Mean change, 95% CI = 2.26, 1.59–2.93) was associated with randomization groups (p = 0.03) but not program adherence (p = 0.09)(Univariate analysis) (Table 6).

**Table 6:** Factors associated with changes in cognitive scores from baseline to 12th month.

	Cognitive test (Change of score from baseline to 12^th^ month)							
	CMMSE		ADAS-Cog		CVFT		Delayed Recall	
	F	p value	F	p value	F	p value	F	p value
**Age**	12.52	0.001	0.08	0.79	0.0	0.99	12.32	<0.001
**Education (years)**	1.35	0.25	0.14	0.71	1.23	0.27	0.41	0.53
**Baseline MCI group**	3.07	0.08	0.75	0.39	4.45	0.04	41.41	<0.001
**Randomization Group**	0.72	0.54	0.64	0.59	3.15	0.03	0.29	0.83
**Program adherence (%)**	4.84	0.03	14.64	<0.001	2.87	0.09	7.16	0.008
**Mean change from baseline (95% CI)**	-0.21(-0.44, 0.02)		3.04(2.77, 3.31)		2.26(1.59, 2.93)		1.95(1.75, 2.16)	

CMMSE—Cantonese version of the Mini-Mental State Examination; ADAS-Cog—Hong Kong version of the Alzheimer’s Disease Assessment Scale; CVFT—Category Verbal Fluency Test; Baseline MCI group—Mild cognitive impairment, single domain versus multiple domain deficits. Mean change from baseline (negative sign referred to deterioration), Univariate analysis

## Discussion

The present study was one of the few non-pharmacological interventions that evaluated the efficacy of structured lifestyle activity interventions in optimizing cognition in a non-white older community. Our findings suggested that there were no changes in CDR-SOB and instrumental activity of daily living scores after participating in structured lifestyle activities for one year. The primary outcome of the intervention, CDR-SOB, was selected to capture possible changes in global cognition and functioning in participants with MCI. A stabilization of CDR-SOB may reflect possible benefits of structured lifestyle activities. However, as CDR-SOB has a restricted range of measures, the evaluation of improvements in CDR-SOB is limited by ceiling effects in participants with minimal or no cognitive impairment. The lack of changes in CDAD-IADL scores may also be affected by ceiling effects, so that subtle changes in everyday function may not be detected. On the other hand, secondary outcome measures showed improvements in cognitive scores including ADAS-Cog, delayed recall and verbal fluency tests. As these tests are sensitive to early changes in AD, the findings supported the possibility that structured leisure activities may have a role in optimizing cognitive function, which are consistent with other RCTs on physical exercise interventions demonstrating improvements in cognitive performance over trial.[25–28] Contrary to our hypothesis that dual modality CP interventions would exert better cognitive benefits over single modality (C or P) interventions, there are no significant differences in cognitive test performance across different intervention programs. We only found that the CP activities resulted in better improvements in CVFT. In another recent study, combined physical and mental activities, though demonstrating cognitive benefits, did not show superiority over active control intervention.[29] It may be because cognitive enhancement effects of activity intervention may be attributable, at least partially, to non-specific stimulation through social interactions.

On the other hand, it is important to consider if the lack of specific benefits in intervention (CP, P, S) activity is related to logistic factors such as intensity of intervention, adherence, or that there would be no specific cognitive benefits in the activity intervention. In a study of a physical fitness training program for patients with Parkinson’s disease in the Netherlands (ParkFit), while the primary outcome did not reveal any change in overall physical activity with 7 day recall compared with control groups, secondary analyses revealed a greater improvement with intervention in other activity measures.[30] We found that participants with milder cognitive deficit (sdMCI), the CP group achieved better ADAS-Cog, CVFT and delayed recall scores at the study end. While cognitive stimulation and physical exercise might help to booster physiological responses and enhance cognition, a therapeutic window may exist when the responses to intervention could be observed more readily. It is possible that at milder stage of cognitive decline, individuals still possess greater capacity for modulation of cognitive function, and may assume greater benefits with integrated stimulation.

However, the findings should be interpreted in the context of its limitations. While we managed to recruit over 550 participants into the study, the sample size for subgroup analyses was still limited. In interpreting the improvement of cognitive test performance across time, it is important to consider the practice effects and increase familiarity of testing materials as a potential reason for improvement although parallel forms were adopted in all memory tests to minimize this bias. The participants who dropped out from the study also reflected a potential bias on findings. Participants with poorer baseline cognition may have a higher default rate, or poorer adherence, which would influence cognitive outcome. Besides, we did not incorporate biological markers into the study to track physiological changes associated with cognition. Further research exploring the association between physiological responses to different lifestyle interventions may give support to the role of such interventions, which will aid the planning of personalized advice on adjuvant life style interventions. Also, one-year observation period is relatively short for dementia prevention. In addition, we assumed three times per week as a minimal standard, and found that adherence was associated with cognitive outcomes. However, we were not able to evaluate the cognitive effects with different frequencies of activity.

We did not aim to design a novel or sophisticated experimental cognitive or physical exercise paradigm. Instead, the present study explored if a pragmatic approach of structured lifestyle activity schedule would benefit people at risk of developing dementia. Our results showed that this approach was both practical and feasible. About 76% of the participants completed this one year study and 73% had satisfactory adherence. The relatively satisfactory completion rate and adherence to the program might be due to that this study was carried out in community social facilities with activity schedules already endorsed by the local older population. Such arrangement would improve its acceptability and user-friendliness to older people with existing cognitive problems.

Among our study participants, age and education did not affect program adherence. On the contrary, women and participants with better baseline cognition appeared to have a higher adherence. This suggested that gender and cognitive abilities are factors that may influence program adherence. The integrated CP group had the lowest adherence rate (65%). More demanding intervention may lead to higher dropout rates, despite greater potential benefits. After controlling for baseline factors that might influence study outcomes, adherence was associated with changes in scores of ADAS-Cog, delayed recall and MMSE. This suggested that adherence is an important factor in determining improvement associated with activity intervention for cognitive health. In people with existing cognitive impairment, special considerations such as regular reminder and encouragement would be important in enforcing good adherence to optimize benefits.

In summary, the present study showed that a 12-month structured lifestyle activity intervention conducted in neighborhood social facilities for older adults with MCI was associated with improvements in cognitive test performance over time, albeit with no change in everyday functioning. In older adults at early stage of observable cognitive deficits, health advice may incorporate regular participation of different lifestyle activities. Lifestyle activity interventions are low cost, low risk and readily adaptable to local culture. Its potential to serve as health promotion strategy in the developing regions where dementia epidemic is expecting and health care costs are challenging should further be explored. For clinical advice to older adults seeking help to improve cognition, our study provided preliminary evidence for ‘Regular practice of cognitive activities and physical exercise in a supportive social environment should start at the earliest possible time AND adherence is the key.’

## Supporting Information

S1 Appendix (DOCX)

S1 CONSORT Checklist CONSORT checklist. (DOCX)

S1 Database (XLS)

S1 Ethics Approval (PDF)

S1 Protocol Trial protocol. (PDF)
