# Supplementary material for: Would Older Adults with Mild Cognitive Impairment Adhere to and Benefit from a Structured Lifestyle Activity Intervention to Enhance Cognition?: A Cluster Randomized Controlled Trial
Source: PLoS One. 2015 Mar 31;10(3):e0118173. doi: 10.1371/journal.pone.0118173 (PMC4380493; doi:10.1371/journal.pone.0118173)
Supplement: S1 Appendix — (DOCX) [file pone.0118173.s001.docx]

**Appendix - Classification & examples of leisure activities**

| **Cognitive** | Reading books, newspapers, or magazine, Using computer or surfing the Internet, Playing board games, Playing mahjong, Playing card games, Gambling, Investment or following the stock market, Participating in forums or discussions, Writing, Calligraphy, Painting, Handicraft, for example, knitting and needlework, Playing a musical instrument | |
| --- | --- | --- |
| **Social** | Attending an interest class, Going to theatres or concerts, Meeting relatives or friends, Watching Drama or Chinese opera performance, Singing with friends, Attending religious activities in church | |
| **Physical** | Mind-body exercise | Tai chi, Qigong, Yoga, Other Chinese-style mind-body exercise |
|  | Aerobic exercise | Chinese martial arts, Jogging or running, Stair climbing, Swimming, Hiking or excursions, Bicycling or using exercise machines, Playing ballgames or racquet sports, Callisthenics, Dancing |
|  | Stretching and toning exercise | Slow walking, pebble trail walking, general stretching and toning exercise |
