# Supplementary material for: Would Older Adults with Mild Cognitive Impairment Adhere to and Benefit from a Structured Lifestyle Activity Intervention to Enhance Cognition?: A Cluster Randomized Controlled Trial
Source: PLoS One. 2015 Mar 31;10(3):e0118173. doi: 10.1371/journal.pone.0118173 (PMC4380493; doi:10.1371/journal.pone.0118173)
Supplement: S1 Ethics Approval — (PDF) [file pone.0118173.s004.pdf]

香港中文大學醫學院
Faculty Of Medicine
The Chinese University Of Hong Kong

醫院管理局
新界東醫院聯網
Hospital Authority
New Territories East Cluster

Joint Chinese University of Hong Kong-New Territories East Cluster Clinical Research Ethics Committee
香港中文大學-新界東醫院聯網 臨床研究倫理 聯席委員會

Flat 3C, Block B, Staff Quarters, Prince of Wales Hospital, Shatin, HK
Tel : (852) 2632 3935 / 2144 5926 Fax : (852) 2646 6653 Website : http://www.crec.cuhk.edu.hk

To: Prof. Linda Chiu Wa LAM (Principal Investigator)
Dept. of Psychiatry
Tai Po Hospital

5 MAY '11

Ethics Approval of Research Protocol

CREC Ref. No.:	**CRE-2011.222-T**
Date of Approval:	**03 May 2011***
Study Title:	**A randomized controlled trial of leisure activity intervention on preservation of cognitive and everyday function in Chinese older adults with early cognitive decline**
Investigator(s):	**Linda Chiu Wa LAM and Ada FUNG**

I write to inform you that ethics approval has been given for you to conduct the captioned study in accordance with the following document(s) submitted:

- Research Proposal
- Participant Information Sheet and Informed Consent Form, Chinese Version

This ethics approval* will be valid for 12 months. Application for further renewal can be made by the submission of the Ethics Renewal and Research Progress Report Form to the CREC (Download the electronic form template from the http://www.crec.cuhk.edu.hk or http://ntec.home/Research%20Ethics/main.asp). You are kindly requested to report to the Committee upon completion of the study.

The Joint CUHK-NTEC Clinical Research Ethics Committee is organized and operated according to ICH-GCP and the applicable laws and regulations.

Miss Winkie Lui
CREC Officer
Joint CUHK-NTEC
Clinical Research Ethics Committee

Encl.
WL/ci

26467287

THE CHINESE UNIVERSITY OF HONG KONG

M E M O

To	:	Prof. LAM Chiu Wa, Linda<br>Department of Psychiatry
From	:	Secretary<br>Survey and Behavioural Research Ethics Committee (SBREC)
Tel.	:	2609 6238
Date	:	28 Mar 2011

Survey and Behavioural Research Ethics SBRE - 43-11

I write to inform you that the SBREC has granted approval to you for conducting the following research:

Project Title	:	A randomized controlled trial of leisure activity intervention on preservation of cognitive and everyday function in Chinese older adults with early cognitive decline
Source of Funding	:	Simon KY Lee Fund for the Elderly
Reference, if any	:	Nil

Thank you for your attention.

Sulan Wong

c.c. Panel Secretary concerned
Director, Research Administration Office
