# Supplementary material for: Would Older Adults with Mild Cognitive Impairment Adhere to and Benefit from a Structured Lifestyle Activity Intervention to Enhance Cognition?: A Cluster Randomized Controlled Trial
Source: PLoS One. 2015 Mar 31;10(3):e0118173. doi: 10.1371/journal.pone.0118173 (PMC4380493; doi:10.1371/journal.pone.0118173)
Supplement: S1 Protocol — (PDF) [file pone.0118173.s005.pdf]

Proposal Title -

A randomized controlled trial of leisure activity intervention on preservation of cognitive and everyday function in Chinese older adults with early cognitive decline

Principal investigator -

Linda Chiu Wa LAM

Professor, Department of Psychiatry, The Chinese University of Hong Kong

Correspondence –

G/F, Multi-centre, Tai Po Hospital, Tai Po, New Territories, Hong Kong

Email: cwlam@cuhk.edu.hk; Fax:26675464; Tel:26076026/26076040

A randomized controlled trial of leisure activities intervention on preservation of cognitive and everyday function in Chinese older adults with early cognitive decline

Abstract

Background: Epidemiologic studies report benefits of cognitive and physical activities on preservation of cognition in older adults. Animal studies suggested that physiological and molecular changes that booster brain reserve are possible mechanisms relating the beneficial effects. In a recent study, we identified that physical activity, especially mind body exercise (Tai Chi), offered benefits in cognitive function. For generalization of the findings into everyday activity schedule for the older community, it is important to evaluate the optimal mode of leisure activity profile for protecting cognitive decline at late life.

Objectives: The proposed study aims to compare the efficacy of different activity schedules in preservation of cognition in older adults at very mild stages of cognitive decline. The main objectives are 1) to evaluate if regular participation of leisure activity will help to maintain cognitive and everyday function, and 2) to identify the optimal profile of leisure activities.

Methods: This will be a Single Blind Randomized Controlled Trial of 12 months duration. Chinese adults (aged 60 or above) with mild cognitive decline (Mild Cognitive Impairment [MCI] and pre-MCI) will be randomized into four intervention groups with 150 subjects per arm. The intervention groups comprised of cognitive (C), physical (P), cognitive-physical (CP) and social (S) programs. Each participant will participate in regular activities arranged by the participating elderly centres. Cognitive and functional performance will be assessed at the baseline, 4th, 8th and 12th months. Cognitive and functional changes would be compared between the intervention groups.

Potential Significance: The findings will generate information on effect sizes of leisure activity intervention for dementia prevention in the Hong Kong Chinese older population. It would provide evidence based information for recommendation as a public health promotion strategy.

Background

Population aging is a global health challenge. Advanced age is associated with high prevalence of cognitive impairment and dementia. A Delphi consensus study projected that the population suffering from dementia in China and West Pacific Region will increase by 300% in the next two decades (1). In Hong Kong, over 10% of the population aged 70 years or above suffers from clinical dementia (2). The relatively low literacy, limited complexity at work and midlife vascular risks in the emerging older cohort suggest a high likelihood of increasing prevalence of cognitive impairment in this locality.

Clinical manifestations of dementia are modulated by complex interactions of environmental and physical factors (3-6). Despite a huge increase in the understanding of basic physiological mechanisms in dementia, treatment efficacy remains limited. Repeated failures of large scale clinical trials suggested that the complex neurodegenerative disorder will not be adequately managed by a single facet approach (7-8).

Lifestyle factors influence cognition. A higher level of participation in physical and cognitive activity participation offer beneficial effects in cognition and protect against dementia (9-11). While physical exercise appears to confer better benefits on cognition, there has not been a consensus as to the dose and modality of physical exercise for optimal neuroprotection. For non-physical leisure activities, cognitive stimulating activities are also reported to assume beneficial role in preserving cognition at late life (12-13). Clinical trials reported benefits of physical exercise on cognitive function, more consistently with attention, processing speed and executive function (11). There were also reports to suggest that computerized cognitive training program offered greater improvements over control groups in memory and attention (14).

Despite that healthy lifestyles are found to be associated with better brain health at late life, there are important issues which need to be further determined. First, lifestyle alterations are hard to sustain, especially if the activities are considered to be demanding and alien to routine schedule in elders. Second, despite evidence to suggest beneficial effects of physical and cognitive activities, the optimal combination and intensity of activities for preservation of cognitive function is not clear. Third, lifestyle intervention should be considered as adjuvant

measure for prevention of dementia. There is a need to carefully select the outcome indicators and determine its respective effect sizes in maintaining cognition.

The present proposal is prepared with reference to the above issues. The aim of this study is to develop a multi-modality leisure activity package that may offer benefits for maintenance of cognitive and everyday functioning in older adults in Hong Kong. The package will be derived from familiar leisure activities that are easily accessible and acceptable. Different combination of activity profiles would be examined for the clinical effectiveness in preserving or enhancing cognition and function, with outcome indicators specially selected for dementia prevention studies in the Chinese community.

Research Objectives and Hypotheses

For the present study, only people with Mild Cognitive Impairment (MCI) and pre-MCI would be recruited. It is hoped that lifestyle intervention offered at this stage will assume greater modulating effects in preventing clinical impairments of dementia to emerge. MCI refers to a condition when subjects presented with subjective or objective cognitive impairment not diagnosable as dementia, but is a recognized risk condition for deterioration. The term pre-MCI refers to pre-symptomatic states when neurodegeneration is at the early stage so that with no obvious clinical deficits are observed (15-16).

The comparative efficacy of 4 leisure activity intervention schedule would be examined with a randomized controlled trial (RCT). The intervention groups comprised of Cognitive (C), Physical (P), Cognitive-Physical (CP) and Social-only (S) programs.

Hypotheses

1. We hypothesize that the CP approach would offer superior effects on cognitive function over single approach (C, P), which will be better than the S approach.
2. We also hypothesize the CP approach will offer the best effect on maintenance of everyday functioning than C and P approach.

Research Plan and Methodology

Research Plan

This study will adopt a single blind randomized control trial design of 12 month duration.

Recruitment centres and participants

Participants will be recruited at the service centres for elders through three NGOs selected by the Simon KY Lee Fund for the Elderly (HOH, BOK, SKH). Interested participants will undergo an eligibility assessment before recruitment. Inclusion criteria are: 1. Subjects 60 years or above. 2. Satisfied criteria for MCI by a cognitive screening package 3. Physically fit with low risks of fall as assessed by the research team.

The exclusion criteria were: 1. Clinical dementia with a Clinical Dementia Rating, CDR >=1(17); 2. Already been prescribed with anti-dementia medication.

Ethical considerations

Participants of this project should be of no major cognitive deficits, and be mentally capable for consent for research. A trained psychiatrist familiar with mental capacity assessment will solicit written informed consent from each participant before eligibility assessment. Ethics approval will be obtained before commencement of study. The protocol will be registered with the CCT Clinical Trials Registry of the Chinese University of Hong Kong and linked to the ChiCTR, WHO-ICTRP China Primary Registry.

Intervention and Group Assignment

The subjects will be randomized into four groups. Appendix showed a classification of leisure activities commonly practiced by older people in Hong Kong. They were categorized into

cognitive, physical, social and recreational in nature by focused groups of occupational therapists, social workers, psychiatric nurses, old age psychiatrists and healthy elders.

The format of intervention would be in groups organized at the social centres. For each intervention group, the following schedule would be adopted.

1. The Social (S) group will serve as the controlled arm. Groups randomized as S would comprise of social activities selected from Appendix (18). For any week, at least 3 social groups of one hour duration should be arranged for the participants.
2. The Cognitive (C) group will be trained with cognitive stimulating activities. Groups randomized as C would comprise of cognitive activities selected from Appendix 2. For any week, at least 3 cognitive groups of one hour duration each should be arranged.
3. The Physical (P) group will have training on physical exercise. Groups randomized as P would comprise of physical activities selected from Appendix. In order to capture the benefits of different modalities of physical exercise, participants will be arranged with physical exercise program with 1 type of stretching & toning exercise, 1 type of mind body exercise and 1 type of aerobic exercise for one hour duration in any one week.
4. The Cognitive-Physical (CP) group will have training on both physical exercise and cognitive activities. Groups assigned as CP would comprise of 2 types of cognitive activities with 1 type of mind body physical exercise. Alternatively, the activity combination may be consisted of 1 type of cognitive activities with 2 types of mind body physical exercise. The activity list will be selected by the appropriate categories as stipulated in Appendix 2. For any week, at least 3 activity groups of one hour duration each should be arranged.

Intervention schedule

For each group, the centre will provide training and facilities for programs at least once per day with frequency three times per week during the one year intervention period. Participants are also encouraged to carry out activities at home at their leisure. A log book will be used to keep track of the compliance. The centre staffs will keep track of attendance and phone up participants every two weeks, participants who do not follow the schedule will be actively followed up.

Randomization procedure & Assessment schedule

Each participating centre will be assigned a special code generated by the computer. The randomization will be kept by the PI and a research assistant. The assessor for clinical outcomes would be blinded to the randomization status. The research staffs who perform the intervention would not know the assessment results. After the eligibility assessment by cognitive screening, cognitive and functional assessments would be conducted at the baseline (after completion of informed consent), at 4th, 8th and 12th months.

Clinical outcome indicators and assessment

Primary Outcome

1. Clinical Dementia Rating (CDR) sum of boxes scores (17). CDR is a semi-structured clinical interview for assessment of global cognitive ability. Six areas are assessed to give an overall rating of global cognition (0, not demented; 0.5, very mild dementia; 1 to 3, mild to severe dementia). The sum of boxes refers to summative scores of the six domains of everyday cognition and functioning measured in CDR (orientation, memory, judgment, habits and hobbies, self care and community activities). It is sensitive to capture functional deterioration of dementia at the earliest changes.

Secondary Outcome

1. Cognitive assessment included the list learning test, digit span, visual span, category verbal fluency (CVFT) and trail making tests (19).
2. Subjective cognitive complaints (SCC) were determined by the Memory Inventory for the Chinese (MIC). It is a 22 item questionnaire exploring subjective cognitive problems in everyday activities (20).

3. Everyday functioning would be assessed by the Disability Assessment for Dementia (DAD), the validated Chinese version assesses both basic and instrumental activities of daily living along three dimensions (initiation, organization and effectiveness). Earlier studies conducted in Hong Kong suggested that the initiation and organization aspects of IADL may be affected in Chinese elders with MCI (21).
4. Motivational symptoms were assessed by the Cornell Scale for depression in dementia (CSDD) and the Neuropsychiatric Inventory (NPI)(22-23).

Sample Size Estimation and Statistical Analyses

The sample size was calculated from the changes in CDR sum of boxes scores in the subgroup analysis of subjects with mild cognitive impairment who also participated in a clinical trial of physical exercise intervention. After one year, there was significant difference in CDR sum of boxes scores in the mind body exercise group. The mean CDR sum of boxes scores was 0.15(0.32) and 0.45(0.71) in exercise and control groups, 60 subjects per MCI and pre-MCI groups will achieve a power of 84% with an alpha of 0.05. Assuming a 25% dropout rate in one year, 75 MCI and 75 pre-MCI subjects will need to be recruited in each arm. A total of 600 subjects will need to be recruited for the present study (24).

Cognitive function changes will be compared between the intervention groups. The mean change of cognitive efficacy parameters from baseline visit to endpoint in each group is compared by paired-samples t-test. For continuous efficacy measures, repeated measures analysis of variance (ANOVA) will be used to compare outcome parameters in different interventions. Intention–to-treat analysis will be carried out with mixed effect models employed to compare group change in each variable across time points from baseline. Adjustments for confounders of age, gender and educational level would be made. ANOVA will be used to compare group differences at the baseline and different time points. Data will be analysis using SPSS with p value set at $p<0.05$. Bonferroni corrections would be made for multiple comparisons.

Existing facilities

The Department of Psychiatry at the Chinese University of Hong Kong will provide technical and statistical support for the study.

Potential for Implementation of Results

Leisure Activities constitute a significant part of daily activity schedule for the older people. It would be of practical health implications if the leisure activity profiles are protective against cognitive decline. The current randomized controlled trials offered information about the potential effectiveness and effect size of preservation of cognition by a multi-modality intervention for older adults at risk of cognitive decline. The findings, if affirmative, will help to guide the implementation of activity intervention for the older community. From the perspective of dementia prevention, it may help to delineate a direction for low-cost low-risk strategies that are feasible and applicable in the Chinese community. From a scientific perspective, the results will also help to deepen understanding on the clinical implications of enhancing cognitive reserve in neurodegenerative conditions.

References

1. Ferri CP, Prince M, Brayne C, Brodaty H, Fratiglioni L, Ganguli M, Hall K, Hasegawa K, Hendrie H, Huang Y, Jorm A, Mathers C, Menezes PR, Rimmer E, Scazufca M; Alzheimer's Disease International. Global prevalence of dementia: a Delphi consensus study. Lancet. 2005;366:2112-7.
2. Lam LC, Tam CW, Lui VW, Chan WC, Chan SS, Wong S, Wong A, Tham MK, Ho KS, Chan WM, Chiu HF. Prevalence of very mild and mild dementia in community-dwelling older Chinese people in Hong Kong. Int Psychogeriatr. 2008;20:135-48.
3. Roe CM, Xiong C, Miller JP, Morris JC. Education and Alzheimer disease without dementia: support for the cognitive reserve hypothesis. Neurology. 2007;68:223-8.
4. Potter GG, Helms MJ, Plassman BL. Associations of job demands and intelligence with cognitive performance among men in late life. Neurology. 2008;70:1803-8.

5. Hall CB, Lipton RB, Sliwinski M, Katz MJ, Derby CA, Verghese J. Cognitive activities delay onset of memory decline in persons who develop dementia. Neurology. 2009;73:356-61.
6. Akbaraly TN, Portet F, Fustinoni S, Dartigues JF, Artero S, Rouaud O, Touchon J, Ritchie K, Berr C. Leisure activities and the risk of dementia in the elderly: results from the Three-City Study. Neurology. 2009;73:854-61.
7. Jones RW. Dimebon disappointment. Alzheimers Res Ther. 2010 Sep 13;2:25. [Epub ahead of print]
8. Lilly Halts Development of Semagacestat for Alzheimer's Disease Based on Preliminary Results of Phase III Clinical. Trialshttp://newsroom.lilly.com/releasedetail.cfm?releaseid=499794
9. Wilson RS, Barnes LL, Aggarwal NT, Boyle PA, Hebert LE, Mendes de Leon CF, Evans DA. Cognitive activity and the cognitive morbidity of Alzheimer disease. Neurology. 2010, 14;75:990-6.
10. Schwenk M, Zieschang T, Oster P, Hauer K. Dual-task performances can be improved in patients with dementia: a randomized controlled trial. Neurology. 2010;74:1961-8.
11. Lam LC, Tam CW, Lui VW, Chan WC, Chan SS, Chiu HF, Wong A, Tham MK, Ho KS, Chan WM. Modality of physical exercise and cognitive function in Hong Kong older Chinese community. Int J Geriatr Psychiatry. 2009;24:48-53.
12. Smith GE, Housen P, Yaffe K, Ruff R, Kennison RF, Mahncke HW, Zelinski EM. A cognitive training program based on principles of brain plasticity: results from the Improvement in Memory with Plasticity-based Adaptive Cognitive Training (IMPACT) study. J Am Geriatr Soc. 2009;57:594-603.
13. Eckroth-Bucher M, Siberski J. Preserving cognition through an integrated cognitive stimulation and training program. Am J Alzheimers Dis Other Demen. 2009;24:234-45.
14. Smith PJ, Blumenthal JA, Hoffman BM, Cooper H, Strauman TA, Welsh-Bohmer K, Browndyke JN, Sherwood A. Aerobic exercise and neurocognitive performance: a meta-analytic review of randomized controlled trials. Psychosom Med. 2010;72:239-52.
15. Petersen RC. Mild cognitive impairment as a diagnostic entity. J Intern Med. 2004;256:183-194.
16. Storandt M, Grant EA, Miller JP, Morris JC. Longitudinal course and neuropathologic outcomes in original vs revised MCI and in pre-MCI. Neurology. 2006 Aug 8;67:467-73.

17. Morris JC: Clinical dementia rating: a reliable and valid diagnostic and staging measure for dementia of the Alzheimer type. Int Psychogeriatr 1997;9 Suppl 1:173-176.
18. Leung GT, Fung AW, Tam CW, Lui VW, Chiu HF, Chan WM, Lam LC. Examining the association between participation in late-life leisure activities and cognitive function in community-dwelling elderly Chinese in Hong Kong. Int Psychogeriatr. 2010;22:2-13.
19. Lam LC, Chau RC, Wong BM, et al. Interim follow-up of a randomized controlled trial comparing Chinese style mind body (Tai Chi) and stretching exercises on cognitive function in subjects at risk of progressive cognitive decline. Int J Geriatr Psychiatry, in press.
20. Lui VW, Lam LC, Chiu HF. Validation of a memory inventory for the assessment of awareness of memory deficits in Alzheimer's disease in Chinese elderly. Int J Geriatr Psychiatry. 2006 ;21:917-23.
21. Mok CC, Siu AM, Chan WC, Yeung KM, Pan PC, Li SW. Functional disabilities profile of chinese elderly people with Alzheimer's disease - a validation study on the chinese version of the disability assessment for dementia. Dement Geriatr Cogn Disord. 2005;20:112-9
22. Cummings JL, Mega M, Gray K, Rosenberg-Thompson S, Carusi DA, Gornbein J. The Neuropsychiatric Inventory: comprehensive assessment of psychopathology in dementia. Neurology 1994; 44: 2308-14.
23. Lam CK, Lim PP, Low BL, Ng LL, Chiam PC, Sahadevan S. Depression in dementia: a comparative and validation study of four brief scales in the elderly Chinese. Int J Geriatr Psychiatry 2004; 19: 422-28.
24. Borenstein M, Rothstein H, Cohen J, Schoenfeld D, Berlin J and Lakatos E. Power and Precision 2 – a computer program for statistical power analysis and confidence intervals. Biostat.

Prepared by

Linda Chiu-wa LAM

MB ChB(CUHK), MD(CUHK), FRCPsych, FHKCPsych, FHKAM(Psychiatry)

January 2010

Classification of leisure activities

**Cognitive**	Reading books, newspapers, or magazines	
	Using computer or surfing the Internet	
	Playing board games	
	Playing mahjong	
	Playing card games	
	Gambling	
	Investment or following the stock market	
	Participating in forums or discussions	
	Writing	
	Calligraphy	
	Painting	
	Handicraft, for example, knitting and needlework	
	Playing a musical instrument	
**Social**	Attending an interest class	
	Joining a social centre	
	Participating in volunteer work	
	Going to museums, exhibitions, theatres or concerts	
	Meeting relatives or friends	
	Drama or Chinese opera performance	
	Singing	
	Attending religious activities	
**Recreational**	Watching television	
	Listening to the radio	
	Listening to music	
	Shopping	
	Cooking for pleasure	
	Fishing	
	Keeping plants	
	Keeping pets	
	Facials or massage	
**Physical**	Mind-body exercise	Tai chi
		Qigong
		Yoga
		Other Chinese-style mind-body exercise

Strenuous aerobic exercise	Chinese martial arts<br>Jogging or running<br>Stair climbing<br>Swimming<br>Hiking or excursions<br>Bicycling or using exercise machines<br>Playing ballgames or racquet sports<br>Callisthenics<br>Dancing
Stretching and toning exercise	Slow walking, pebble trail walking, general stretching and toning exercise
